# Refined estimates of local recurrence risks by DCIS score adjusting for clinicopathological features: a combined analysis of ECOG-ACRIN E5194 and Ontario DCIS cohort studies

**DOI:** 10.1007/s10549-018-4693-2

**Published:** 2018-01-31

**Authors:** E. Rakovitch, R. Gray, F. L. Baehner, R. Sutradhar, M. Crager, S. Gu, S. Nofech-Mozes, S. S. Badve, W. Hanna, L. L. Hughes, W. C. Wood, N. E. Davidson, L. Paszat, S. Shak, J. A. Sparano, L. J. Solin

**Affiliations:** 1 Department of Radiation Oncology, Sunnybrook Health Sciences Centre, Toronto, ON M4N 3M5 Canada; 2 Institute for Clinical Evaluative Sciences, Toronto, ON Canada; 3 Sunnybrook Health Sciences Centre, University of Toronto, Toronto, ON Canada; 4 Dana-Farber Cancer Institute, Boston, MA USA; 5 Genomic Health Incorporated, Redwood City, CA USA; 6 University of California, San Francisco (UCSF), San Francisco, CA USA; 7 Department of Pathology, Sunnybrook Health Sciences Centre, Toronto, ON Canada; 8 Departments of Pathology and Internal Medicine, Clarian Pathology Laboratory of Indiana University, Indianapolis, IN USA; 9 Harris Radiation Therapy Center at Gordon Hospital, Calhoun, GA USA; 10 Emory University, Atlanta, GA USA; 11 University of Pittsburgh, Pittsburgh, PA USA; 12 Montefiore Medical Center, Bronx, NY USA; 13 Department of Radiation Oncology, University of Pennsylvania, Philadelphia, PA USA; 14 Department of Radiation Oncology, Albert Einstein Healthcare Network, Philadelphia, PA USA

**Keywords:** Ductal carcinoma in situ, DCIS, Meta-analysis, Prognosis, Local recurrence, Genomic

## Abstract

**Purpose:**

Better tools are needed to estimate local recurrence (LR) risk after breast-conserving surgery (BCS) for DCIS. The DCIS score (DS) was validated as a predictor of LR in E5194 and Ontario DCIS cohort (ODC) after BCS. We combined data from E5194 and ODC adjusting for clinicopathological factors to provide refined estimates of the 10-year risk of LR after treatment by BCS alone.

**Methods:**

Data from E5194 and ODC were combined. Patients with positive margins or multifocality were excluded. Identical Cox regression models were fit for each study. Patient-specific meta-analysis was used to calculate precision-weighted estimates of 10-year LR risk by DS, age, tumor size and year of diagnosis.

**Results:**

The combined cohort includes 773 patients. The DS and age at diagnosis, tumor size and year of diagnosis provided independent prognostic information on the 10-year LR risk (*p* ≤ 0.009). Hazard ratios from E5194 and ODC cohorts were similar for the DS (2.48, 1.95 per 50 units), tumor size ≤ 1 versus  > 1–2.5 cm (1.45, 1.47), age ≥ 50 versus < 50 year (0.61, 0.84) and year ≥ 2000 (0.67, 0.49). Utilization of DS combined with tumor size and age at diagnosis predicted more women with very low (≤ 8%) or higher (> 15%) 10-year LR risk after BCS alone compared to utilization of DS alone or clinicopathological factors alone.

**Conclusions:**

The combined analysis provides refined estimates of 10-year LR risk after BCS for DCIS. Adding information on tumor size and age at diagnosis to the DS adjusting for year of diagnosis provides improved LR risk estimates to guide treatment decision making.

**Electronic supplementary material:**

The online version of this article (10.1007/s10549-018-4693-2) contains supplementary material, which is available to authorized users.

## Introduction

Ductal carcinoma in situ (DCIS) is a noninvasive breast cancer but some women will go on and develop invasive breast cancer [1]. Our inability to elucidate which DCIS lesions will progress to invasion and which ones will remain indolent culminate in recommendations that women with DCIS undergo treatment. Most women will be treated by breast-conserving surgery (BCS) followed by the administration of whole breast radiotherapy (RT), which has been proven to lower the risk of local recurrence (LR) (DCIS or invasive) after BCS [2]. Subset analyses from randomized trials demonstrate a similar relative (50%) reduction in LR risk with RT, but the absolute benefit from RT is not uniform for all patients. Some women will derive no or a very small absolute benefit from RT, resulting in unnecessary exposure to radiation and its potential toxicities (over-treatment), while in others the omission of RT may result in a higher risk of LR (and invasive LR) that might have been avoided by treatment (under-treatment) [3]. To reduce over-treatment and under-treatment of DCIS, ascertainment of more precise estimates of individualized LR risk after BCS is desirable to help clinicians and patients more accurately assess the risks of LR with the potential absolute benefits of treatment.

The Oncotype DCIS score (DS) is a 12-gene expression assay based on the Oncotype DX Recurrence score [4]. The DS reports a numeric value ranging from 0 to 100 and a categorical risk group: low risk (0–38), intermediate risk (39–54) and high risk (55–100). The ECOG-ACRIN E5194 (E5194) prospective cohort study initially reported the significance of the DS as an independent predictor of LR in selected women treated by BCS alone [5–7]. More recently, the DS was validated as a predictor of LR in the Ontario population-based DCIS cohort [8]. Multivariable analyses from both studies found that in addition to the DS, age at diagnosis and tumor size were also significant predictors of LR; however, current estimates of local and invasive LR risks associated with the DS do not adjust for these effects.

The objective of this analysis is to combine the data from the E5194 (with extended 12-year follow-up data) and Ontario cohorts to provide refined and more precise estimates of recurrence risk after BCS alone for DCIS. We performed a patient-specific meta-analysis [9] to evaluate the impact of the DS alone, age at diagnosis and tumor size alone or integration of all three parameters, on the predicted 10-year risks of LR and invasive LR. In addition, we report 10-year local and invasive recurrence risk estimates for each DS risk group (low, intermediate, high) adjusting for the effects of age, tumor size and year of diagnosis to provide more accurate estimates of recurrence risk to aid treatment decision making following BCS for DCIS.

## Methods

### Patient cohorts

#### ECOG-ACRIN E5194 cohort

The ECOG-ACRIN E5194 study was a prospective, non-randomized clinical trial [5, 6]. There were two cohorts of patients: (1) low- or intermediate-grade DCIS, tumor size 2.5 cm or smaller (Cohort 1; *N* = 561); or (2) high-grade DCIS, tumor size 1 cm or smaller (Cohort 2; *N* = 104). Treatment for all patients included surgical excision (lumpectomy) of the primary DCIS tumor with a minimum negative margin width ≥ 3 mm or no tumor on re-excision. Radiation treatment was not allowed. The study was amended in May 2000 to allow adjuvant tamoxifen as optional.

#### Ontario DCIS cohort

**Fig. 1:**
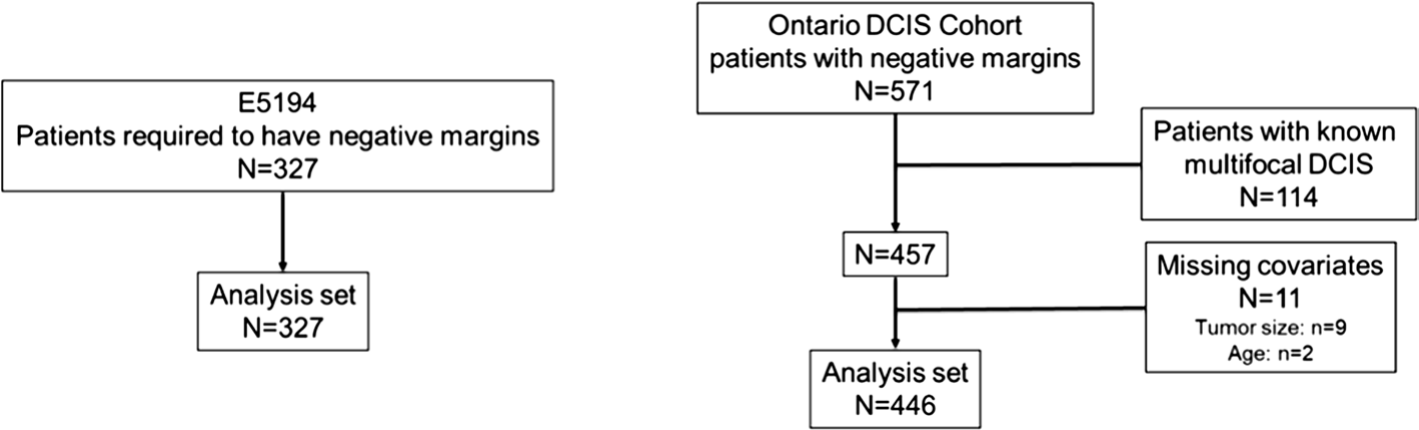
Study cohort

The methods used to establish the Ontario DCIS cohort have been previously described [8, 10]. The population cohort includes 3303 cases with pure DCIS treated by BCS; 1658 treated by BCS alone and 1662 by BCS + RT. We obtained tissue blocks in 1751 cases (*N* = 828, BCS alone; *N* = 923, BCS + RT); we calculated the DS in 571 cases treated by BCS alone with clear margins. Patients with missing tumor size had their tumor size imputed [11]. The study cohort for this analysis includes 446 individuals with pure DCIS treated by BCS alone (Fig. 1).

For both the E5194 and Ontario cohort studies, systematic, pre-defined pathology review was performed [12].

#### Patient-specific meta-analysis

Fixed-effects patient-specific meta-analysis [9] was used to determine the risk of any LR using information from the two DS studies. Based on a Cox proportional hazards framework, we estimated the patient-specific log cumulative hazard for each study, combined the estimates of the log cumulative hazard across studies by weighting each study estimate by the inverse of its variance and then conducted the appropriate transformation to derive the risk estimate. This methodology is based on the assumption that study cohorts are equal in their underlying level of risk, once differences in the characteristics of the cohort have been accounted for. For each study, we present hazard ratios (HR) (95% confidence intervals (CI)), the 10-year LR risk estimate for patients in the two cohorts and the average estimated risk within each DS group (low, intermediate, high), tumor size and age category, along with the minimum and maximum risk possible values for DS results in each group.

The statistical significance of the independent contributions of the DS and the clinicopathological covariates tumor size, age at diagnosis and year of surgery to the LR risk assessment was evaluated using meta-analysis likelihood ratio tests that summed the likelihood ratio Chi-square statistics and their degrees of freedom across E5194 and Ontario cohort studies. The clinical significance of integrating DS result with tumor size and age in risk estimation for patients with year of surgery 2000 or later compared to DS result alone and to tumor size and age alone was evaluated using predictiveness curves [13] and, in particular, the proportion of combined cohort patients with estimated 10-year LR risk ≤ 8, ≤ 10, and > 15%.

## Results

### Study cohort

The study cohort includes 327 patients from E5194 and 446 patients from the Ontario cohort (*N* = 773). The median age (range) at diagnosis was 61 years (10th–90th percentile 45–77) in E5194 cohort and 62 years (45–79) in the Ontario cohort. In E5194 cohort, tumor size was ≤ 1 cm in 260 (79.5%) patients, the median DS was 25 and the median follow-up interval was 11.5 years (range: 0.2–15.9). In the Ontario cohort, 181 (40.6%) of cases had tumor size ≤ 1 cm, the median DS was 30 and median follow-up interval was 9.8 years (range: 0.1–16.2). The proportion of patients with low- , intermediate- and high-risk scores were 70.3, 16.2 and 13.5% in E5194 and 64.6, 16.1 and 19.3% in the Ontario cohort. Among 327 patients in the E5194 cohort, there were 53 LRs (27 invasive LRs). Among 446 patients in the Ontario DCIS cohort, there were 65 LRs (38 invasive LRs) (Table 1).

**Table 1:** Patient characteristics

	E5194 (*n* = 327)	Ontario cohort (*n* = 446)
Age at diagnosis		
Median (10th–90th percentile)	61 (45–77)	62 (45–79)
< 50 years	66 (20.2%)	92 (20.6%)
≥ 50 years	261 (79.8%)	354 (79.4%)
Tumor size		
≤ 1 cm	260 (79.5%)	181 (40.6%)
> 1–2.5 cm	67 (20.5%)	238 (53.4%)
> 2.5 cm	0	27 (6.1%)
Nuclear grade		
Low	29 (8.9%)	50 (11.2%)
Intermediate	187 (57.2%)	267 (59.9%)
High	111 (33.9%)	129 (28.9%)
DCIS score median (10th–90th percentile)	25 (8–58)	30 (5–63)
DCIS score risk group		
Low risk	230 (70.3%)	288 (64.6%)
Intermediate risk	53 (16.2%)	72 (16.1%)
High risk	44 (13.5%)	86 (19.3%)
Year of diagnosis		
1994–1999	147 (45.0%)	255 (57.2%)
2000 or later	180 (55.0%)	191 (42.8%)

### Multivariable analyses

#### Any local recurrence

Multivariable Cox models were fit separately to the data from each cohort. There was no significant difference in the (Breslow) baseline cumulative hazard estimate at 10 years between the two cohorts (Wald test *p* = 0.49). Furthermore, there was no significant difference in the HRs associated with each covariate between the two cohorts (*p* = 0.86) allowing for the two datasets to be combined for analysis.

In the E5194 cohort, an increase of 50 units in the DS (DS/50) was associated with a 2.5-fold increased hazard of LR (HR 2.48; 95% CI 1.29, 4.75). There was an increase in the hazard of LR associated with tumor size > 1–2.5 cm compared to ≤ 1 cm (HR 1.45, 95% CI 0.78, 2.69), and decreased hazard associated with age ≥ 50 years at diagnosis (HR 0.61, 95% CI 0.33, 1.11) and year of diagnosis 2000 or later (HR 0.67, 95% CI 0.38, 1.17), although these effects did not achieve statistical significance. In the Ontario cohort, an increase in DS/50 was associated with a twofold increased hazard for LR (HR 1.95; 95% CI 1.14, 3.32). The hazard ratio for LR associated with tumor size > 1–2.5 cm was 1.47 (95% CI 0.82, 2.64) and 2.99 (95% CI 1.32, 6.76) for tumor size ≥ 2.5 cm compared to ≤ 1 cm. We observed decreased risks of LR associated with age ≥ 50 years at diagnosis (HR 0.61, 95% CI 0.33, 1.11) and year of diagnosis 2000 or beyond (HR 0.49, 95% CI 0.28, 0.87) (Table 2).

**Table 2:** Multivariable Cox models for any local recurrence

Effect	ECOG 5194		Ontario DCIS cohort	
	Hazard ratio (95% CI)	*p* value	Hazard ratio (95% CI)	*p* value
DCIS score/50	2.48 (1.29, 4.75)	0.006	1.95 (1.14, 3.32)	0.014
Tumor size (cm)				
> 1–2.5 versus ≤ 1	1.45 (0.78, 2.69)	0.24	1.47 (0.82, 2.64)	0.19
> 2.5 versus ≤ 1	–	–	2.99 (1.32, 6.76)	0.009
Age at diagnosis (years)				
≥ 50 versus < 50	0.61 (0.33, 1.11)	0.10	0.84 (0.48, 1.49)	0.55
Year of diagnosis				
2000 or later versus before 1999	0.67 (0.38, 1.17)	0.16	0.49 (0.28, 0.87)	0.016

High nuclear grade was not significantly associated with the risk of LR in the E5194 (HR 0.64; 95% CI 0.33, 1.23, *p* = 0.18) or the Ontario (HR 0.98; 95% CI 0.55, 1.75, *p* = 0.95) cohorts (*p* = 0.40) (Table S-1). There were no significant two-way interactions among the covariates (*p* = 0.68).

#### Invasive local recurrence

A smaller number of covariates were used in the model for invasive local recurrence, in part because the number of invasive events was smaller (*N* = 65). Data for tumor size > 2.5 cm were only available for the Ontario cohort tumor size ≤ 2.5 cm and was not associated with an increased hazard of invasive LR (E5194 HR 0.91, 95% CI 0.34–2.42; Ontario HR 1.17, 95% CI 0.56–2.46). Accordingly, models for invasive LR include dichotomized tumor size (> 2.5 cm vs. ≤ 2.5 cm), the DS (DS/50 units), and year of diagnosis (2000 or later vs. before 2000) were fit separately to each cohort. In the E5194 cohort, an increase of DS/50 was associated with a 3.02-fold (95% CI 1.28, 7.14) increased hazard of invasive LR and diagnosis in year 2000 or later was associated with a decreased hazard of invasive LR (HR 0.92, 95% CI: 0.42,2.00). In the Ontario cohort, an increase in DS/50 (HR 2.18, 95% CI: 1.12, 4.27) and tumor size > 2.5 cm (HR 2.20, 95% CI 0.85, 5.65) were associated with an increased hazard of invasive LR. Although large tumor size did not achieve statistical significance, the magnitude of the HR was considered too large to ignore and as such was included in the prediction models. Year of diagnosis 2000 or later was associated with a decrease in the hazard of invasive LR (HR 0.57, 95% CI 0.28, 1.20) (Table 3).

**Table 3:** Multivariate Cox proportional hazards regression model for the development of invasive local recurrence after breast-conserving surgery alone

	E5194		Ontario DCIS cohort	
Effect	HR (95% CI)	*p* value	HR (95% CI)	*p* value
DCIS score/50	3.02 (1.28, 7.14)	0.012	2.18 (1.12, 4.27)	0.023
Tumor size				
> 2.5 versus ≤ 2.5 cm	N/A		2.20 (0.85, 5.65)	0.10
Diagnosis in 2000 or later	0.92 (0.42, 2.00)	0.83	0.57 (0.28, 1.20)	0.14

#### Predicting local recurrence risk

The model that included the DS, tumor size and age at diagnosis and year of diagnosis demonstrated improved prediction of the 10-year risk of LR compared to a model based on the DS alone (*p* = 0.009) or one based solely on tumor size, age at diagnosis and year of diagnosis without the DS (*p* = 0.002). This indicates that the DS and the clinicopathological covariates each contribute independent prognostic information on the estimated 10-year risk of LR after BCS.

To assess and compare the clinical utility of each model (DS alone, tumor size and age at diagnosis alone or integration of all three parameters combined), we examined its ability to identify patients with a low estimated 10-year risk of LR (defined as 10-year LR risk ≤ 8%) and its ability to identify those with an estimated higher risk of LR (defined as 10-year LR risk > 15%) after treatment by BCS alone. We found that integrating the effects of the DS, tumor size and age at diagnosis identified a greater proportion of cases with a low risk of LR compared to models based on the DS alone, or one based solely on tumor size and age at diagnosis alone (all models were adjusted for the effect of year of diagnosis of 2000 or later). The integration of all three covariates (DS, tumor size and age at diagnosis) identified 25.9% of women with an estimated 10-year risk LR ≤ 8% compared to 17.7% of cases based on the DS alone; the model based on tumor size and age at diagnosis alone did not identify any patients with a 10-year risk of LR ≤ 8% after BCS alone.

**Fig. 2:**
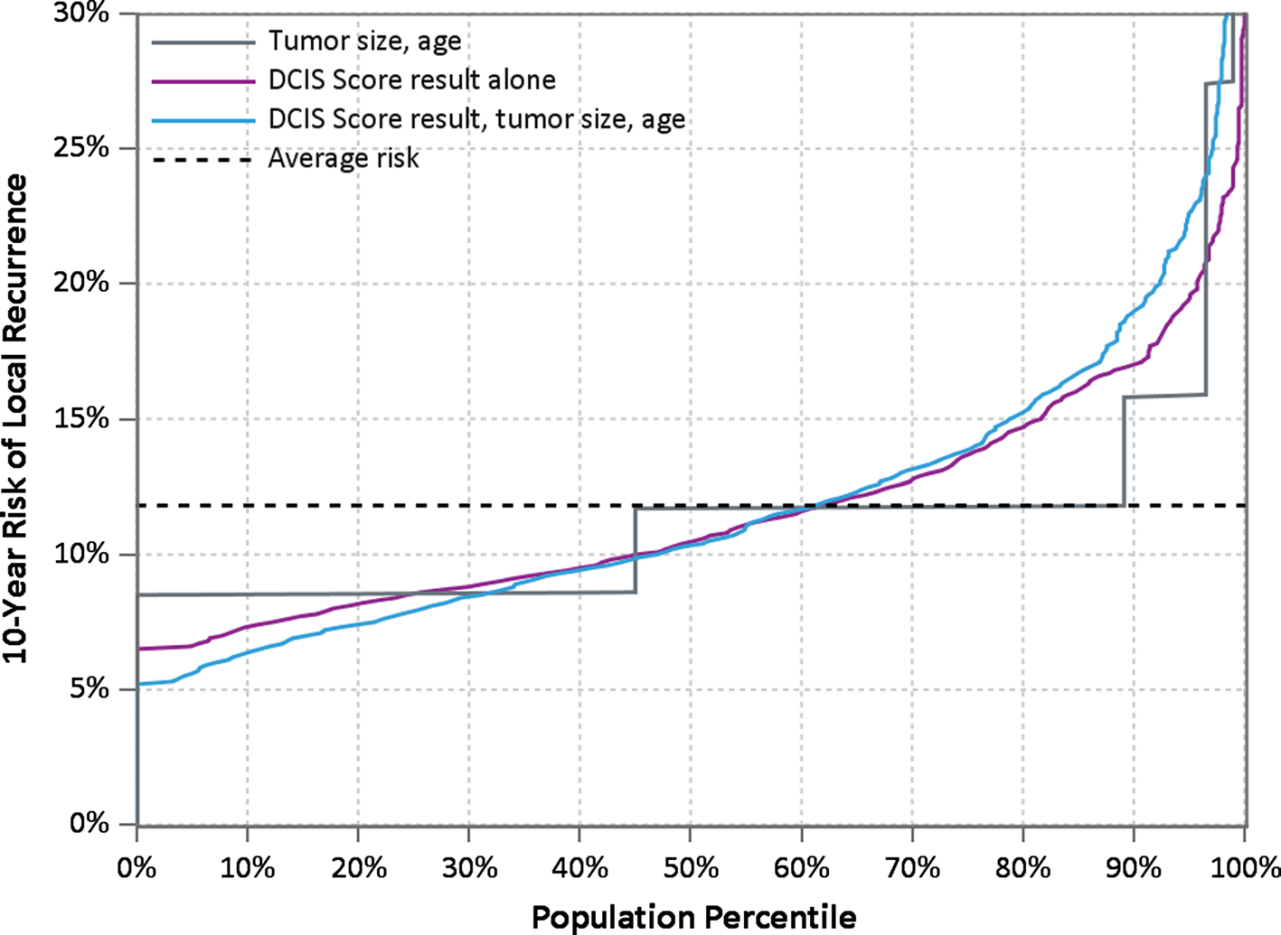
Predicted 10-year risk of local recurrence after breast-conserving surgery alone for DCIS: a comparison of models based on the DCIS score, tumor size and age at diagnosis. The estimated 10-year risk of LR after BCS alone against the proportion of patients with this risk or less is shown. The model integrating the effects of the DCIS score, tumor size and age at diagnosis identified more women with low and high risks of LR compared to models based on the DS alone or tumor size and age at diagnosis alone

In addition, and importantly, the model integrating the effects of tumor size and age at diagnosis with the DS also identified more women (21.1%) with a predicted high risk of LR (defined as 10-year LR risk > 15%) compared to models based on the DS alone (which identified 18.4% of women at high risk of LR) or one based on tumor size and age at diagnosis alone (which identified only 10.9% of women at high risk of LR) (Table 4). Predictiveness curves plotting the estimated 10-year risk of LR after BCS alone against the proportion of patients with this risk or less are shown in Fig. 2. The curves suggest improved discrimination between high-risk and low-risk patients in the population with utilization of DS combined with age and tumor size.

**Table 4:** Proportion of patients with predicted 10-year LR risk ≤ 8 or > 15%

Model	Proportion of cases and predicted 10-year risk of LR		
	≤ 8%	≤ 10%	> 15%
DCIS score, tumor size, age combined	25.9%	47.0%	21.1%
DCIS score	17.7%	45.1%	18.4%
Tumor size, age	0	44.1%	10.9%

#### Refined estimates of recurrence risk: combining the effects of age at diagnosis, tumor size and year of diagnosis with the DCIS score

Table 5 summarizes the estimated average 10-year risks of LR following BCS alone based on age at diagnosis, tumor size and DCIS risk group (risk estimates at the low and high DS limits of each risk group are also provided), adjusted for year of diagnosis 2000–2003. This demonstrates the impact of the DS on the risk of LR by age and tumor size. For example, for women age ≥ 50 years with tumor size 1.1–2.5 cm and an intermediate risk DS, the average 10-year risk of LR is 13.9%, ranging from 12.8% for those with a DS of 39 to 15.6% for those with a DS of 54. Overall, women age ≥ 50 years with tumor size ≤ 1 cm and a low-risk DS have an average 10-year risk of LR of 7.2% (ranging from 5.3 to 10.0%) and those ≥ 50 years with tumors 1.1–2.5 cm have an average 10-year risk of LR of 10.1% (ranging from 7.3 to 12.6%). For the same age and tumor size groups, a high-risk DS was associated with approximately a twofold increased risk of LR at 10 years compared to cases with a low-risk DS. Overall, women age ≥ 50 years with tumor size ≤ 1 cm and a high-risk DS have an average 10-year risk of LR of 14.6% (ranging from 12.9 to 23.1%) and those ≥ 50 years with tumors 1.1–2.5 cm have an average 10-year risk of LR of 19.5% (ranging from 15.8 to 28.7%).

**Table 5:** 10-year risks of any local recurrence (DCIS or invasive) after breast-conserving surgery alone by combinations of age, tumor size and DCIS score

10-year risk of local recurrence (%)^a^ (range^b^) by DCIS score group				
Tumor size (cm)	Age (year)	Low DCIS score (0–38)	Intermediate DCIS score (39–54)	High DCIS score (55–100)
≤ 1	≥ 50	7.2 (5.3–10.0)	11.3 (10.2–12.7)	14.6 (12.9–23.1)
	< 50	10.2 (7.4–13.9)	15.8 (14.1–17.4)	19.6 (17.7–30.7)
1.1–2.5	≥ 50	10.1 (7.3–12.6)	13.9 (12.8–15.6)	19.5 (15.8–28.7)
	< 50	14.5 (10.1–17.2)	18.9 (17.4–21.1)	23.2 (21.4–37.2)
> 2.5	≥ 50	20.4 (14.9–27.0)	29.1 (27.4–33.3)	41.1 (33.8–54.4)
	< 50	30.2 (20.6–36.1)	39.5 (36.6–43.6)	48.6 (44.1–66.5)

^a^Average risk for E5194 and Ontario DCIS cohort patients in DCIS score groups

^b^Risks at boundaries of DCIS score groups

**Fig. 3:**
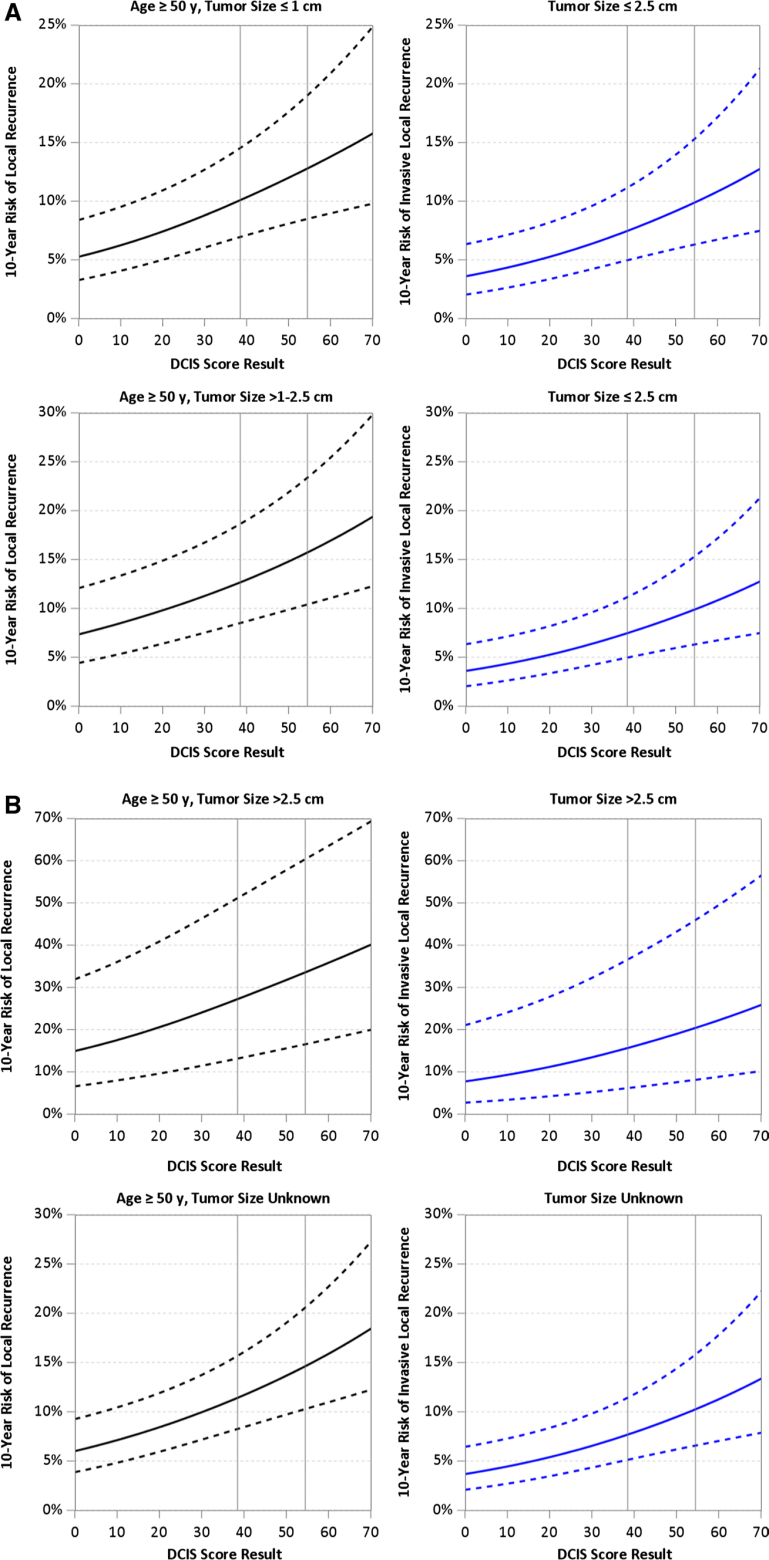

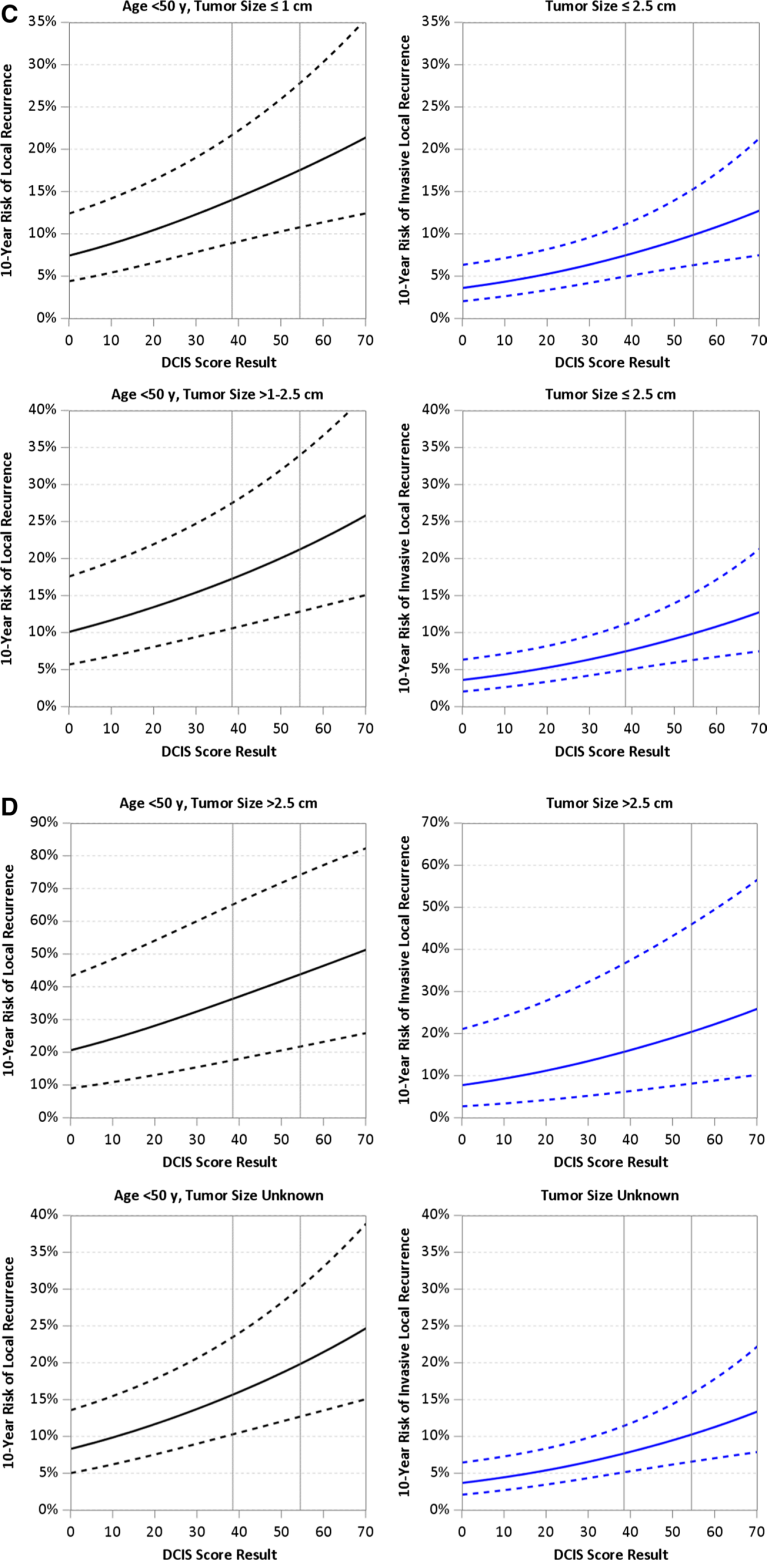
**a** 10-year risk of any local recurrence (left panels) and invasive local recurrence (right panels) estimated from patient-specific meta-analysis, for patients with diagnosis in 2000 or later, age ≥ 50 at diagnosis and tumor size ≤ 2.5 cm. **b** 10-year risk of any local recurrence (left panels) and invasive local recurrence (right panels) estimated from patient-specific meta-analysis, for patients with diagnosis in 2000 or later, age ≥ 50 at diagnosis and tumor size > 2.5 cm or unknown. **c** 10-year risk of any local recurrence (left panels) and invasive local recurrence (right panels) estimated from patient-specific meta-analysis, for patients with diagnosis in 2000 or later, age < 50 at diagnosis and tumor size ≤ 2.5 cm. **d** 10-year risk of any local recurrence (left panels) and invasive local recurrence (right panels) estimated from patient-specific meta-analysis, for patients with diagnosis in 2000 or later, age < 50 at diagnosis and tumor size > 2.5 cm or unknown

For patients with tumor size > 2.5 cm, we produced risk estimates using the HR from the Ontario cohort together with HRs for other factors estimated separately for each cohort [9]. Women with lesions > 2.5 cm had substantially higher estimated 10-year risks of LR after BCS alone ranging from 20.4% for those with a low-risk DS, age ≥ 50–48.6% for women age < 50 with a high-risk DS; however, there were only a few (*N* = 23) young women with a high-risk DS treated by BCS alone. Risk estimates for LR and invasive LR are shown in Fig. 3a–d.

The average estimated 10-year risks of invasive LR for tumor size ≤ 2.5 cm and a low- , intermediate- or high-risk DS were 5.3% (3.6–7.4%), 8.5% (7.5–9.8%) and 12.1% (10.0–20.5%). The corresponding estimates based on tumor size > 2.5 cm and DS risk group were 11.6% (7.7–15.5%), 17.1% (15.8–20.3%) and 26.2% (20.6–40.0%), but these estimates are based on few cases (Table 6).

**Table 6:** 10-year risks of invasive local recurrence after breast-conserving surgery alone by tumor size and DCIS score

10-year risk of invasive local recurrence (%)^a^ (range^b^) by DCIS score group				
Tumor size (cm)	Age (year)	Low DCIS score (0–38)	Intermediate DCIS score (39–54)	High DCIS score (55–100)
≤ 1	≥ 50	5.3 (3.6–7.4)	8.5 (7.5–9.8)	12.1 (10.0–20.5)
	< 50			
1.1–2.5	≥ 50			
	< 50			
> 2.5	≥ 50	11.6 (7.7–15.5)	17.1 (15.8–20.3)	26.2 (20.6–40.0)
	< 50			

^a^ Average risk for E5194 and Ontario DCIS Cohort patients in DCIS Score groups

^b^ Risks at boundaries of DCIS Score groups

## Discussion

This combined analysis provides refined estimates of the 10-year risk of LR and invasive LR of DCIS lesions treated by BCS alone. Integrating the effects of tumor size and age at diagnosis with the DS provides improved prediction and substantially better separation of low-risk from high-risk patients than either DS alone or information based on tumor size and age alone (without the DS).

Treatment decision making relies on estimating an individual’s risk of recurrence after BCS weighed against the potential benefits of treatment. Regression models estimate the relationships among individual variables and the likelihood of developing LR. Predictiveness curves combine the effects of risk modeling with the distribution of the risk-prediction covariates in the patient population [13]. They illustrate the range and distribution of risk estimates within populations and provide a way to compare the performance of different models. We compared the performance of three models in predicting the risk of LR at 10 years after treatment by BCS alone with clear margins using data from the E5194 and Ontario cohorts; one model included the DS alone, one model was based on tumor size, age at diagnosis, and a third model combined all three parameters, adjusting for year of diagnosis. We found that integration of the effects of tumor size, age at diagnosis and year of diagnosis with the DS significantly improves LR risk prediction compared with estimates based on the DS alone or one based on tumor size, age and diagnosis year alone (without the DS).

The extent to which predicted risk estimates will influence treatment decision making relies on the thresholds of LR risk that determine if additional treatment is warranted. In this regard, models that best classify individuals at very low or high risk of recurrence have the greatest clinical utility. We evaluated the ability of each model to predict cases at very low risk of LR (defined as 10-year risk of LR ≤ 8%) or those at high risk (defined as 10-year risk of LR > 15%) after BCS alone. The model integrating tumor size and age at diagnosis with the DS performed better at both extremes. The DS/tumor size/age model identified 25.9% of the cohort as having a 10-year LR risk ≤ 8%. By comparison, the DS alone classified only 17.7% of cases while the model based on tumor size and age alone did not identify any cases as having a 10-year LR risk ≤ 8%. This suggests that the DS adjusted for the effects of tumor size and age at diagnosis can help reduce over-treatment by identifying significantly more women with a very low risk of LR after treatment by BCS alone for whom the benefit of RT would be extremely small. If the threshold for additional treatment is (a 10-year LR risk of) > 10%, then almost half the cases in the cohort (47%) would avoid additional treatment. These are mostly women ≥ 50 years of age with lesions ≤ 1 cm and DS ≤ 38 (68%) but they also include women ≥ 50 years of age with lesions 1–2.5 cm and DS ≤ 21 (23%) and women ≤ 50 years of age with lesions ≤ 1 cm and DS ≤ 17 (9%). Table 4 lists the average refined estimates of LR risk within DS groups and demonstrates the impact of each parameter on the 10-year LR risk by DS risk group. Women ≥ 50 years of age at diagnosis with lesions ≤ 2.5 cm and a low-risk DS or those aged < 50 years at diagnosis and tumor size ≤ 1 cm and a low-risk DS had average estimated 10-year risks of LR < 10.2% following treatment by BCS alone.

In addition, we found that integrating the impact of tumor size and age at diagnosis with the DS performed better at predicting cases with a high risk of LR (> 15%) after BCS alone where additional treatment would be warranted [21.1% compared to 18.4% classified by the DS alone and only 11% classified using tumor size and age without the DS (Fig. 2)]. There were women in all categories of age at diagnosis and lesion size with estimated LR > 15%.

This analysis has several strengths. It was derived from a prospective cohort and a population-based cohort treated by BCS alone with negative margins, includes large numbers of annotated samples with expert pathology assessment and DS molecular testing; therefore, the risk model is generalizable to similar patients in the general population. The baseline risks of LR and the HRs associated with the clinicopathological parameters were similar in the two cohorts (Table 2), indicating it is appropriate to apply a combined analysis. LR risks have declined over time [14]; therefore, adjusting the risk estimates to reflect outcomes beyond year 2000 to provide more accurate prediction of expected outcomes of women treated in the current era.

This analysis has several limitations. The study population includes few women (*N* = 37) with tumors > 2.5 cm treated by BCS alone (6% of Ontario cohort); therefore, risk estimates in women with DCIS lesions > 2.5 cm should be interpreted with caution.

This analysis does not account for the impact of tamoxifen. Approximately one-third of the E5194 and 17% of those > 65 years in Ontario cohort received tamoxifen. Tamoxifen was used more frequently by patients diagnosed in 2000 or later (48.9%) than patients diagnosed before 2000 (15.0%). A sensitivity analysis of E5194 data was conducted to assess the effect of tamoxifen regression parameter estimates. A multivariate model was fit with the DS, tumor size, age, diagnosis year and a time-dependent indicator for tamoxifen use (Table S-2). The values of the HRs are similar to those in the main analysis, indicating that tamoxifen use did not greatly influence the estimates in this study.

In summary, this combined analysis provides refined estimates of the 10-year LR and invasive LR risk after treatment by BCS alone. Integrating the effects of tumor size and age at diagnosis with the DS provides improved prediction and better separation of very low-risk from high-risk patients (Table 6, Fig. 2). Specifically, these refined estimates identify a greater proportion of women with a 10-year LR risk ≤ 8% after BCS alone who could safely avoid additional treatment since the absolute benefit from additional interventions would be low and a greater proportion of women with a higher 10-year LR risk > 15% in whom efficacious treatments are needed to lower the risk of future recurrence. This can improve clinical decision making and the management of DCIS patients by helping clinicians and patients more accurately weigh risk of recurrence with the potential benefits of treatment.

## Electronic supplementary material

Below is the link to the electronic supplementary material.

Supplementary material 1 (DOCX 33 kb)

Supplementary material 2 (DOCX 32 kb)
