# Supplementary material for: Refined estimates of local recurrence risks by DCIS score adjusting for clinicopathological features: a combined analysis of ECOG-ACRIN E5194 and Ontario DCIS cohort studies
Source: Breast Cancer Res Treat. 2018 Jan 31;169(2):359–69. doi: 10.1007/s10549-018-4693-2 (PMC5945747; doi:10.1007/s10549-018-4693-2)
Supplement: Supplementary file 1 — Supplementary material 1 (DOCX 33 kb) [file 10549_2018_4693_MOESM1_ESM.docx]

**Supplementary Table S-1. Multivariate Cox Proportional Hazards Regression Model including nuclear grade for the development of any local recurrence after breast-conserving surgery alone**

|  | **E5194**  **(n=327)** | | **Ontario Cohort**  **(n=446)** | |
| --- | --- | --- | --- | --- |
| Effect | HR (95% CI) | p-value | HR (95% CI) | p-value |
| DCIS Score (per 50 units) | 3.28 (1.51, 7.12) | .003 | 1.97 (1.08, 3.58) | .027 |
| Tumor size (cm) |  |  |  |  |
| >1-2.5 vs. ≤1 cm | 1.43 (0.77, 2.65) | .26 | 1.48 (0.83, 2.65) | .19 |
| > 2.5 vs. ≤1 | ─ | ─ | 3.00 (1.32, 6.80) | .009 |
| Age ≥ 50 y | 0.60 (0.33, 1.09) | .094 | 0.84 (0.48, 1.49) | .55 |
| Diagnosis in 2000 or later | 0.67 (0.38, 1.17) | .15 | 0.49 (0.28, 0.88) | .016 |
| High nuclear grade | 0.64 (0.33, 1.23) | .18 | 0.98 (0.55, 1.75) | .95 |
