# Supplementary material for: Refined estimates of local recurrence risks by DCIS score adjusting for clinicopathological features: a combined analysis of ECOG-ACRIN E5194 and Ontario DCIS cohort studies
Source: Breast Cancer Res Treat. 2018 Jan 31;169(2):359–69. doi: 10.1007/s10549-018-4693-2 (PMC5945747; doi:10.1007/s10549-018-4693-2)
Supplement: Supplementary file 2 — Supplementary material 2 (DOCX 32 kb) [file 10549_2018_4693_MOESM2_ESM.docx]

**Supplementary Table S-2. Sensitivity analysis of E5194 study including time-dependent tamoxifen use in the multivariate Cox proportional hazards regression models for any local recurrence**

| Effect | Hazard Ratio  95% Confidence Interval | p-value |
| --- | --- | --- |
| DCIS Score result  (per 50 units) | 2.47  (1.28, 4.74) | .007 |
| Tumor Size >1-2.5 cm  vs. ≤1 cm | 1.45  (0.78, 2.70) | .24 |
| Age ≥ 50 y | 0.61  (0.33, 1.11) | .11 |
| Diagnosis in 2000 or later | 0.77  (0.42, 1.44) | .42 |
| Tamoxifen use | 0.69  (0.32, 1.47) | .33 |
